# A potential role of the JNK pathway in hyperoxia-induced cell death, myofibroblast transdifferentiation and TGF-β1-mediated injury in the developing murine lung

**DOI:** 10.1186/1471-2121-12-54

**Published:** 2011-12-15

**Authors:** Zhang Li, Rayman Choo-Wing, Huanxing Sun, Angara Sureshbabu, Reiko Sakurai, Virender K Rehan, Vineet Bhandari

**Affiliations:** 1 Division of Perinatal Medicine, Department of Pediatrics, Yale University School of Medicine, New Haven, CT 06520, USA; 2 Division of Neonatology, Department of Pediatrics, Harbor UCLA Medical Center, David Geffen School of Medicine at UCLA, Torrance, CA, 90502, USA; 3 Department of Anesthesiology, Qilu Hospital of Shandong University, No. 107 Wenhua West Road, Lixia District, 250012 Jinan, Shandong, China; 4 Department of Systems Biology, Beth Israel Deaconess Medical Center, Center for Life Sciences, 3 Blackfan Circle, Boston, MA 02115, USA

## Abstract

**Background:**

Transforming growth factor-beta 1 (TGF-β1) has been implicated in hyperoxia-induced cell death and impaired alveolarization in the developing lung. In addition, the c-JunNH2-terminal kinase (JNK) pathway has been shown to have a role for TGF-β1-mediated effects. We hypothesized that the JNK pathway is an important regulator of hyperoxia-induced pulmonary responses in the developing murine lung.

**Results:**

We used cultured human lung epithelial cells, fetal rat lung fibroblasts and a neonatal TGF-β1 transgenic mouse model. We demonstrate that hyperoxia inhibits cell proliferation, activates cell death mediators and causes cell death, and promotes myofibroblast transdifferentiation, in a dose-dependent manner. Except for fibroblast proliferation, the effects were mediated via the JNK pathway. In addition, since we observed increased expression of TGF-β1 by epithelial cells on exposure to hyperoxia, we used a TGF-β1 transgenic mouse model to determine the role of JNK activation in TGF-β1 induced effects on lung development and on exposure to hyperoxia. We noted that, in this model, inhibition of JNK signaling significantly improved the spontaneously impaired alveolarization in room air and decreased mortality on exposure to hyperoxia.

**Conclusions:**

When viewed in combination, these studies demonstrate that hyperoxia-induced cell death, myofibroblast transdifferentiation, TGF-β1- and hyperoxia-mediated pulmonary responses are mediated, at least in part, via signaling through the JNK pathway.

## Background

Hyperoxia exposure to the developing lung is a critical factor in the occurrence of the most common chronic lung disease in neonates, namely bronchopulmonary dysplasia (BPD) [1]. This is especially important given the recent trend in non-invasive ventilation of preterm neonates; hence, bringing the role of hyperoxia (as opposed to endotracheal tube mechanical ventilation) to the forefront among the environmental factors contributing to "new" BPD [1-3]. While efforts have been made to decrease hyperoxia exposure to the developing lung, the incidence of BPD has actually increased [3]. An improved understanding of the mechanisms of hyperoxia-induced cell death and lung injury would be extremely helpful in formulating potential therapeutic strategies with the goal of ameliorating BPD [3,4].

An important step in this direction would be to understand if varying levels of exposure to hyperoxia [5] have differential impact on lung cell death mechanisms, and if so, evaluate potential therapeutic targets. The mitogen-activated protein kinase (MAPK) signal transduction pathways are comprised of at least 3 distinct families; namely, the extra-cellular signal-regulated kinase (ERK), p38, and c-JunNH2-terminal kinase (JNK) pathways [6]. Although the functions of the JNK pathways are not yet fully understood, they are known to regulate cell proliferation, differentiation, death and inflammatory responses [6]. JNK signaling has been implicated in hyperoxia-induced pulmonary injury responses [6-10].

One of the molecular mediators implicated in hyperoxia-induced cell death and impaired alveolarization in the developing lung is transforming growth factor-beta 1 (TGF-β1) [11-16], which has also been associated with human BPD [17]. Another molecule that has been implicated as a downstream mediator of TGF-β1 signaling in the newborn lung is connective tissue growth factor (CTGF) [18]. Recently, the JNK pathway has been implicated for TGF-β1-mediated effects in the developing lung [19].

Hence, we hypothesized that inhibition of the JNK signaling pathway in *in vitro *and *in vivo *models of hyperoxia-exposure to the lung would improve survival. Furthermore, inhibition of the JNK signaling pathway would mitigate TGF-β1- and hyperoxia-mediated effects in the developing lung. Our goal was to study cellular responses on exposure to hyperoxia in the presence of JNK inhibition (JNKi), using cultured human lung epithelial cells and fetal rat lung fibroblasts. In addition, we evaluated the responses of lung-specific TGF-β1 overexpression *in vivo *in the developing lung in the presence of JNKi, with or without hyperoxia. Specifically, we evaluated mortality, cell proliferation, myofibroblast transdifferentiation and markers thereof (i.e. peroxisome proliferator-activated receptor γ (PPARγ), adipocyte differentiation-related protein (ADRP), fibronectin and LEF-1), cell death mediators (FAS, FAS-L, caspase 3), and CTGF expression in our *in vitro *and *in vivo *models. Furthermore, we utilized a newborn (NB) wild type (WT) murine BPD model to assess the impact of JNKi on alveolarization.

## Results

### Hyperoxia-induced A549 cell death and its mediators are dependent on the JNK pathway

**Figure 1:**
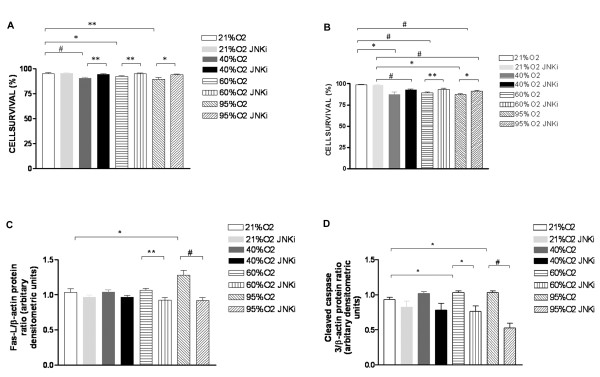
**Effect of JNK inhibition on cell death and it's mediators in hyperoxia-exposed cells**. A549 cells were exposed to 21% up to 95% O_2 _and cell viability (trypan blue) was assessed at 24 h (**1A**). MLE-12 cells were exposed to 21% up to 95% O_2 _and cell viability (TUNEL assay) was assessed at 24 h (**1B**). The noted values represent assessments in a minimum of 4 measurements in each group. Next, A549 cells were exposed to 21% up to 95% O_2 _and FAS, FAS-L, procaspase 3 and cleaved caspase 3 proteins were assessed by Western Blot. Independent experiments were done in the presence of the JNK pathway inhibitor. The ratios of FAS-L and cleaved caspase-3 with β-actin were quantified by densitometery (**1C and 1D**). The figures are illustrative of a minimum of 4 experiments. O_2_: oxygen; JNKi: JNK inhibitor. *P < 0.05, **P ≤ 0.02, #*P *≤ 0.01.

We initially exposed A549 and MLE cells to varying levels of hyperoxia (40%, 60% and 95% O_2_) for 24 h and noted increased cell death, compared to 21% O_2_, at 24 h. Importantly, this effect appeared to be dose-dependent (Figures 1A and 1B; Additional File 1**Table S1**). We also noted increased levels of total and phosphorylated JNK (P-JNK) protein with increasing levels of hyperoxia (Additional File 1**figure S1**). Using the JNK pathway inhibitor SP600125 in a dose of 5 μM decreased phospho-JNK (Additional File 1**figure S1**), and was accompanied by a significant increase in cell viability (Figure 1A and 1B; Additional File 1**Table S1**). We had similar results even when we extended the hyperoxia-exposure period to 48 h (Additional File 1**figure S2**).

To further characterize the mechanism of hyperoxia-induced cell death in the A549 cells, we evaluated FAS, FAS-L, procaspase-3, and cleaved caspase-3. We noted increased FAS-L and cleaved caspase-3 protein, with increasing concentrations of hyperoxia (Figures 1C and 1D; Additional File 1**figure S3A**). Addition of JNKi mitigated this process (Figures 1C and 1D; Additional File 1**figure S3B**). Hence, our data suggests that the hyperoxia-induced molecular signals acting via the JNK pathway could be potential targets for prevention of hyperoxia-induced lung cell death.

### Hyperoxia-induced alveolar interstitial fibroblasts (AIFs) cell proliferation is not dependent on the JNK pathway

**Figure 2:**
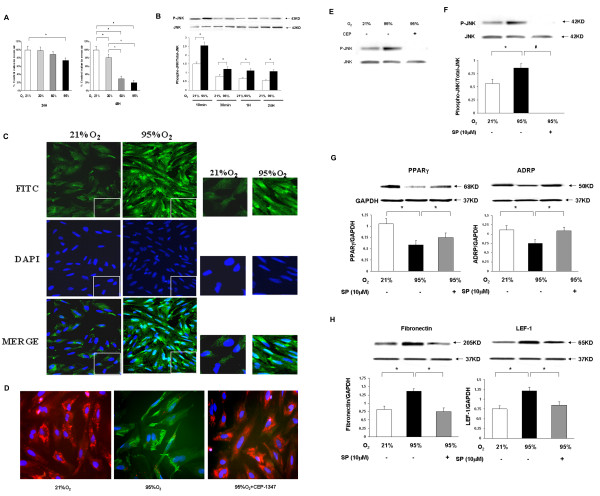
**Effect of JNK inhibition on fetal alveolar interstitial fibroblasts (AIF) in hyperoxia**. AIF were exposed to 21% up to 95% O2 over 24 h and 48 h, and cell proliferation was assessed by thymidine uptake (**2A**). Total and P-JNK were assessed (**2B**) and P-JNK staining was confirmed by immunocytochemistry (**2C**). Use of JNKi (CEP-1347) blocked the hyperoxia-induced decrease in lipid droplet staining (red fluorescence) and an increase in α-SMA staining (green fluorescence), two key markers of AIF-to-MYF transdifferentiation (**2D**) and diminished P-JNK (**2E**). Use of JNKi (SP600125) diminished P-JNK (**2F**) and blocked the hyperoxia-induced decrease in peroxisome proliferator-activated receptor γ (PPARγ) and adipocyte differentiation-related protein (ADRP) levels (**2G**), and the accompanying increase in fibronectin and LEF-1 levels (**2H**). O2: oxygen; JNKi: JNK inhibitor (SP600125 for A549 and AIFs, CEP-1347 for AIFs). All values represent a minimum of 4 measurements in each group and all figures are illustrative of a minimum of 4 experiments. *P < 0.05, **P ≤ 0.02, #*P *≤ 0.01.

We next examined the effect of hyperoxia on AIF proliferation and whether this was modulated via JNK activation. Similar to the effect of hyperoxia on epithelial cells, we noted a significant decrease in AIF proliferation on exposure to hyperoxia, the effect being much more profound at 48 h vs. 24 h (Figure 2A). There was significant JNK activation on exposure to hyperoxia, evident at 10 minutes following exposure to hyperoxia and persisting even at 24 h (Figure 2B). The hyperoxia-induced JNK activation was also corroborated by immunocytochemistry (Figure 2C). In contrast to our observations on epithelial cells, JNKi using SP600125 in a dose of 10 μM did not block hyperoxia-induced decrease in AIF proliferation (data not shown).

### Hyperoxia-induced AIF-to-Myofibroblast (MYF) transdifferentiation is dependent on the JNK pathway

Since hyperoxia induces AIF-to-MYF transdifferentiation [20], we next determined if this process is JNK-dependent. Exposure to AIF to 95% O_2 _for 24 h resulted in a marked decrease (absence) in lipid droplet staining (red fluorescence) with a concomitant marked increase in α-SMA staining (green fluorescence) (Figure 2D), indicating hyperoxia-induced AIF-to-MYF transdifferentiation. Both of these changes were remarkably blocked by concomitant JNKi (1 μM CEP-1347) (Figure 2D), implying JNK's critical role in AIF-to-MYF transdifferentiation. JNKi was confirmed by Western analysis (Figure 2E). Similar results were obtained on using the JNK inhibitor, SP600125 (10 μM) (Figure 2F). Hyperoxia-induced decrease in PPARγ and ADRP levels (Figure 2G), and the accompanying increase in fibronectin and LEF-1 levels (Figure 2H), key indicators of AIF-to-MYF transdifferentiation, were also blocked by concomitant treatment with JNKi, further confirming JNK's role in AIF-to-MYF transdifferentiation.

Hyperoxia-induced epithelial cell death and increased presence of myofibroblasts have been implicated in the pathogenesis of BPD [4,21]. Since TGFβ1 has been recognized to be an important mediator in epithelial cell death and transformation to alveolar myofibroblasts in BPD [3,22], we also evaluated the role of TGFβ1 *in vitro *and in developmentally-appropriate *in vivo *models in relation to hyperoxia and JNK pathway inhibition.

### TGF-β1 and CTGF expression is increased in A549 lung cells on exposure to hyperoxia

**Figure 3:**
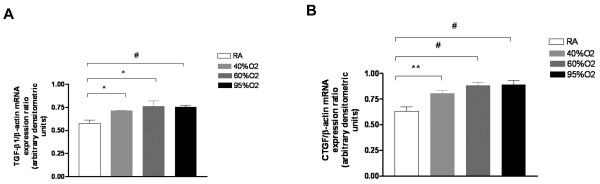
**Effect of hyperoxia on TGF-β1 and CTGF expression**. A549 cells were exposed to 21% up to 95% O_2 _and TGF-β1 and CTGF were assessed by semi-quantitative RT-PCR, and their ratio with β-actin quantified by densitometery (**3A and 3B**). The figures are illustrative of a minimum of 4 experiments. TGF-β1: transforming growth factor beta; CTGF: connective tissue growth factor; O_2_: oxygen. *P < 0.05, **P ≤ 0.02, #*P *≤ 0.01.

Since TGF-β1 and CTGF have been shown to be induced by hyperoxia, we decided to investigate if they were induced in our *in vitro *hyperoxia model. TGF-β1 and CTGF mRNA expression were increased in a dose-dependent manner in A549 cells exposed to varying concentrations of hyperoxia (40%, 60% and 95%) for 24 h (Figures 3A and 3B).

### *In vivo *inhibition of JNK pathway increases survival in hyperoxia-exposed NB WT and TGF-β1 TG mice

**Figure 4:**
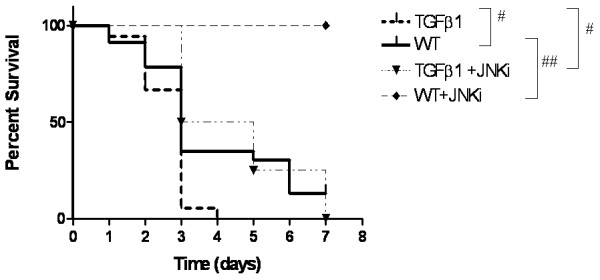
**Survival in hyperoxia**. NB TGF-β1 TG and WT liter-mate control mice on dox water, with or without treatment with JNKi, were exposed to 100% O_2 _and survival was assessed. The noted values represent assessments in a minimum of 4 animals in each group, of 4 independent experiments. WT: wild type; TGF-β1: transforming growth factor beta; JNKi: JNK inhibitor. #*P *≤ 0.01, ##*P *≤ 0.001.

We used hyperoxia-induced acute lung injury model to test if the *in vitro *cell death protective response of the JNKi could improve animal survival *in vivo*. We exposed NB WT and TGF-β1 TG mice on dox water to hyperoxia (100% O_2_), with and without JNKi. NB TGF-β1 TG mice had significantly increased mortality in hyperoxia, compared to litter-mate WT controls (Figure 4). NB WT and TGF-β1 TG, treated with daily injections of the JNKi had significantly increased survival in hyperoxia (Figure 4), compared with their respective controls (WT vs.WT +JNKi, p < 0.0001; TGF-β1 TG vs. TGF-β1 TG +JNKi, p = 0.01). Thus, inhibition of the JNK pathway was protective in terms of survival in the hyperoxia-induced developing lung injury model.

### *In vivo *inhibition of JNK pathway improves alveolarization in NB TGF-β1 TG mice in room air (RA)

**Figure 5:**
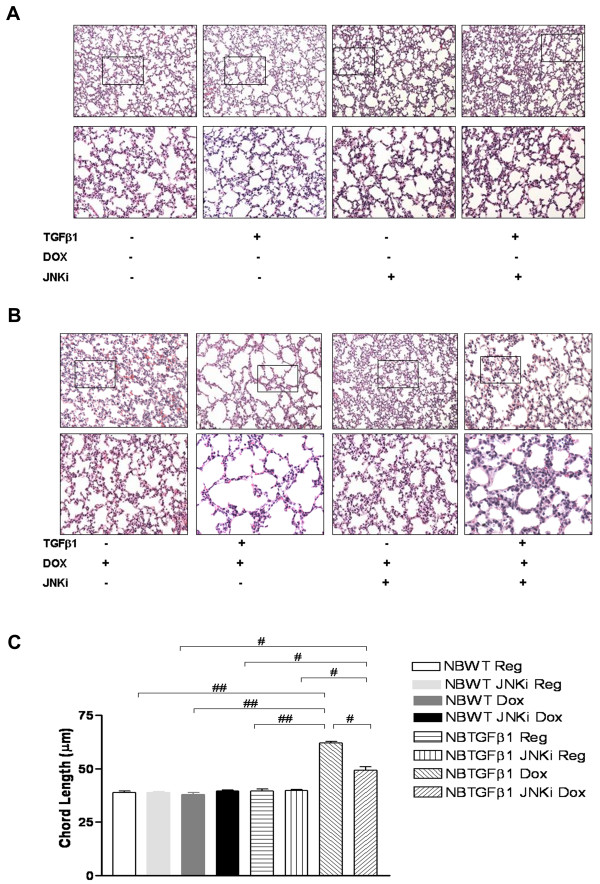
**Effect of JNK inhibition on NB TGF-β1-induced alterations in lung architecture**. NB TGF-β1 TG and WT liter-mate control mice on dox or regular water, with or without treatment with JNKi, were sacrificed at PN10, with activation of TGF-β1 at PN7. H&E staining of lung histology in low (10X; top row) and high (from inset in upper panel; 20X; bottom row) power magnification is shown demonstrating partial rescue of altered alveolar architecture of NB TGF-β1 TG mice lungs with JNKi (**5A and 5B**). The figures are illustrative of a minimum of 4 animals in each group. Alveolar size, as measured by chord length, confirmed the above observations (**5C**). Each bar represents the mean ± SEM of a minimum of three animals. NB: newborn; WT: wild type; TGF-β1: transforming growth factor beta1 transgenic; Reg: regular water; Dox: doxcycline water; JNKi: JNK inhibitor. #*P *≤ 0.01, ##*P *≤ 0.001.

**Figure 6:**
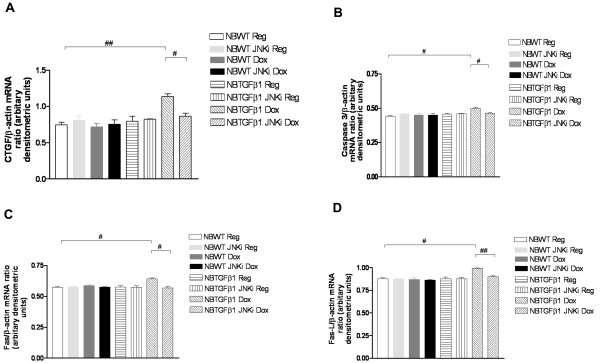
**Effect of JNK inhibition on CTGF and cell death pathway mediators in NB TGF-β1-induced lung injury**. NB TGF-β1 TG and WT liter-mate control mice on dox or regular water, with or without treatment with JNKi, were sacrificed at PN10, with activation of TGF-β1 at PN7. mRNA expression of CTGF, caspase 3, FAS, and FAS-L were assessed. The noted values represent assessments in a minimum of 3 animals in each group. Independent experiments were done in the presence of the JNK pathway inhibitor. The ratios of CTGF, caspase 3, FAS, and FAS-L with β-actin were quantified by densitometery (**6A-D**). The figures are illustrative of a minimum of 3 experiments. NB: newborn; WT: wild type; CTGF: connective tissue growth factor; TGF-β1: transforming growth factor beta1 transgenic; Reg: regular water; Dox: doxcycline water; JNKi: JNK inhibitor. #*P *≤ 0.01, ##*P *≤ 0.001.

Earlier reports have highlighted the role of the TGF-β signaling pathway in alveolarization of the developing lung. Specifically, the NB TGF-β1 TG mouse lung has been shown to have impaired alveolarization in RA [13]. To assess the impact of inhibition of the JNK pathway in this process, we treated with daily injections of the JNK inhibitor, NB TGF-β1 TG mice on dox water from PN7 to PN10, along with littermate WT controls. As noted in Figures 5A and 5B, use of JNKi, in the presence of TGF-β1 activation, improved alveolarization. However, the phenotype was only partially rescued, as evidenced by lung morphometry measurements (Figure 5C). We also confirmed that total and P-JNK protein were increased with TGF-β1 activation and were decreased in the mice lung tissue when treated with JNKi (data not shown). To assess if these changes were secondary to mediators of the cell death pathway, we evaluated the mRNA expression of caspase 3, FAS, and FAS-L, in these lungs. We noted increased caspase 3, FAS, and FAS-L, with exposure to TGF-β1 along with increased expression of CTGF mRNA in the NB lungs on TGF-β1 activation. Addition of JNKi blocked this process. We confirmed our observations by quantification of the expression of CTGF, caspase-3, FAS and FAS-L, as noted in Figures 6A, B, C, and 6D, respectively. Thus, TGF-β1-induced impaired alveolarization in RA is mediated, at least in part, by cell death pathway regulators acting via the JNK pathway in this *in vivo *model.

### *In vivo *inhibition of JNK pathway did not impact on BAL fluid TGF-β1 levels in NB TGF-β1 TG mice in room air

**Figure 7:**
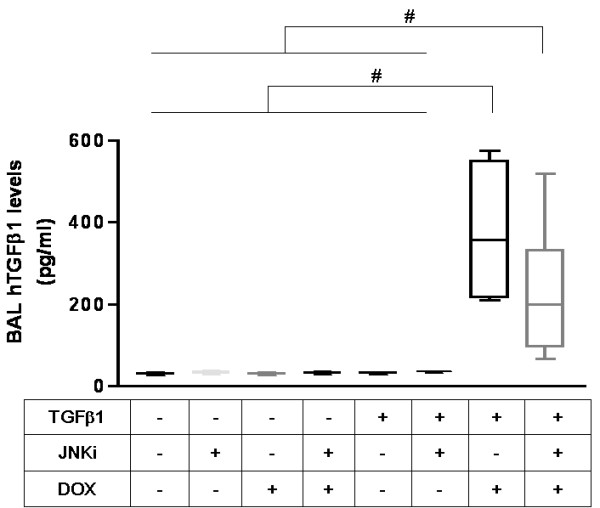
**Effect of JNK inhibition on human TGF-β1 BAL fluid levels**. NB TGF-β1 TG and WT liter-mate control mice on dox or regular water, with or without treatment with JNKi, were sacrificed at PN10, with activation of TGF-β1 at PN7. Bronchoalveolar lavage fluid concentrations of human TGF-β1 were measured. The noted values represent assessments in a minimum of 3 animals in each group. NB: newborn; WT: wild type; TGF-β1: transforming growth factor beta1 transgenic; Reg: regular water; Dox: doxcycline water; JNKi: JNK inhibitor; BAL: bronchoalveolar lavage fluid; h: human. #*P *≤ 0.02.

JNK pathway inhibition could alter TGF-β1-induced effects in the lung by altering the production of TGF-β1 or altering its effector capacity. To clarify this, we compared the levels of human TGF-β1 in BAL fluids from WT and TG mice treated with JNKi on regular or dox water, in RA. As can be seen in Figure 7 similar levels of BAL TGF-β1 were seen in TG mice with or without treatment with JNKi, while on dox water, in RA. These measurements demonstrate that interventions that inhibit JNK pathway act by altering the TGF-β1 effector pathway (but not TGF-β1 levels), in our model.

### *In vivo *inhibition of JNK pathway improves alveolarization in NB WT murine BPD model

**Figure 8:**
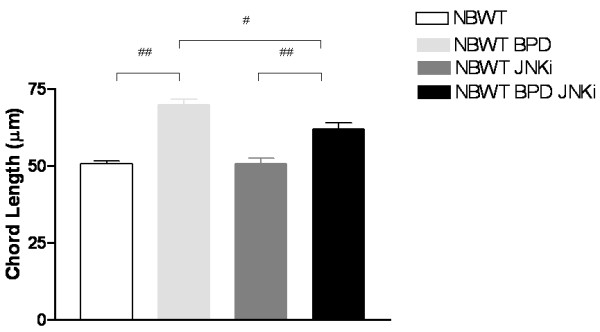
**Effect of JNK inhibition on lung architecture in the NB WT BPD murine model**. NB WT BPD murine model (as described in Methods) and control mice on regular water, with or without treatment with JNKi, were sacrificed at PN14. Alveolar size, as measured by chord length, noted a significant improvement in lung alveolarization in the BPD model, with JNK inhibition. Each bar represents the mean ± SEM of a minimum of four animals. NB: newborn; WT: wild type; BPD: bronchopulmonary dysplasia; JNKi: JNK inhibitor. #*P *≤ 0.01, ##*P *≤ 0.001.

As expected, the murine BPD mice lungs had large, simplified alveoli with significantly increased chord lengths (Figure 8). Interestingly, inhibition of JNK pathway improved alveolarization (Figure 8). However, as evidenced by lung morphometry, the lung phenotype was only partially rescued. This suggests that hyperoxia-induced impaired alveolarization is mediated, at least in part, by regulators acting via the JNK pathway in this *in vivo *model, with one of these regulators being TGF-β1.

## Discussion

Studies were undertaken to test the hypothesis that the JNK pathway is an important regulator of hyperoxia-induced pulmonary responses. These studies demonstrate that hyperoxia inhibits cell proliferation, stimulates cell death, and alters myofibroblast transdifferentiation in a dose-dependent manner. These effects are significantly impacted by inhibiting the JNK pathway. Furthermore, there is increased expression of TGF-β1 and CTGF, on exposure to hyperoxia, both of which are known to signal via the JNK pathway. Our studies also demonstrate that hyperoxia-induced mortality and alveolarization are improved when the JNK pathway is inhibited, in the presence of excess TGF-β1 and CTGF, in the developing lung. When viewed in combination, these studies demonstrate that hyperoxia-induced cell death and TGF-β1-mediated pulmonary responses are mediated via signaling, at least in part, through the JNK pathway.

In our evaluation of varying concentrations of hyperoxia on cell death, all 3 concentrations of hyperoxia significantly reduced cell survival, compared to RA. We, however, could not discern any significant differences between the 3 concentrations of O_2 _on cell death. Use of JNKi was able to restore cell survival to RA values, in all 3 O_2 _concentrations. MLE-12 cells exposed to 95% O_2 _had increased cell survival on inhibition of the JNK pathway [7,23]. Using siRNA against JNK1 in A549 cells exposed to 95% O_2 _decreased interleukin-8 expression, a pro-inflammatory cytokine [8]. Data on cell viability were not reported in that study [8]. Our data supports the contention that JNK pathway inhibition has a significant protective response in lung epithelial cells exposed to 95% O_2_. In addition, we report the novel finding that such an effect is also noted at lower concentrations (40% and 60%) of O_2 _in our *in vitro *model.

While hyperoxia-exposed decrease in cell proliferation in AIF was not impacted upon by JNKi, in line with the findings of Hashimoto et al in human lung fibroblasts, we found that AIF to MYF transdifferentiation was blocked by inhibiting JNK activation [24].

Our studies show that the death-receptor pathway and the executioner caspase 3 are involved in the process of hyperoxia-induced epithelial cell death. These data are supported by an earlier report using MLE-12 cells exposed to 95% O_2 _[25]. In addition, we also report the novel observation that JNKi also impacts FAS-L and caspase-3 protein in A549 lung epithelial cells exposed to 95% O_2_.

To begin to understand the *in vivo *relevance of our findings, we selected TGF-β1 as our cytokine of interest for a variety of reasons. Firstly, the JNK pathway has been implicated in TGF-β1 signaling in lung cells [19,24,26-29], specifically CTGF [19,26,28] and cell death [19,27] as well as myofibroblast transformation [24]. Secondly, hyperoxia has been shown to upregulate TGF-β1 in premature rat lungs [30]. Hence, we first confirmed increased expression of TGF-β1 and CTGF in our *in vitro *model, before proceeding to test our hypothesis in the NB TGF-β1 TG mice.

In the hyperoxia-induced acute lung injury model, JNKi administration was significantly protective in terms of survival in NB TGF-β1 TG mice. Importantly, the survival of the NB WT mice treated with JNKi was 100% after 7 days of 100% O_2 _exposure. This suggests that non-TGF-β1-dependent, but hyperoxia-induced molecular mediators signaling via the JNK pathway, are also involved.

Since TGF-β1 has been implicated in BPD, we used the lung-specific overexpression model to evaluate the impact of JNKi on alveolarization. We selected PN7 as the starting point as the mouse lung is in the alveolar phase at this time, and hyperoxia-induction of TGF-β1 was noted then [30]. We used a 3-day treatment duration, as it takes about 48 hours for the TGF-β1 induction by dox to be sustained in our TG model, as previously described [13]. Expectedly, as previously described [13], activation of TGF-β1 resulted in impaired alveolarization. Inhibition of the JNK pathway was able to improve this to a significant extent, compared to appropriate controls (NB WT mice on regular or dox water, and TGF-β1 on regular water). The improvement in alveolar architecture, however, was only partially corrected to appropriate control levels (NB WT mice on regular or dox water, and TGF-β1 on regular water, all treated with JNKi), as noted in Figure 5. This could be reflective of the short duration of treatment with JNKi that was employed.

It is important to point out that JNKi did not significantly alter the TGF-β1 levels in the BAL fluid of the TG mice on dox water (Figure 7). Hence, the effects noted above with JNKi were due to effects downstream of TGF-β1 pathway activation.

To further assess the potential for clinical translation, we used the NB WT murine BPD model and found significant, but partial, improvement in lung morphometry with JNK pathway inhibition.

Interestingly, a recent publication has reported lung-targeted conditional overexpression of CTGF to have a phenotype of BPD [31]. This data supports our findings of increased CTGF on TGF-β1 activation in the NB lung, which also has a phenotype of BPD [13]. Importantly, we noted decreased expression of CTGF and cell death pathway regulators with JNK pathway inhibition association with improvement of the BPD phenotype in the NB TGF-β1 TG mice lungs.

In addition to inhibiting JNK, SP600125 also inhibits ERK 1/2. The evaluation of the role of ERK 1/2 in our modeling systems was beyond the scope of the present manuscript. There are few limitations of our study: First, while A549 cells mimic lung epithelial cells, it is a transformed cell line, and hence, may not mimic the effects of freshly-isolated lung epithelial cells or *in vivo*. Second, the use of different strains of mice, at variable gestational ages, and different doses of JNK inhibitors can lead to significantly different results. To illustrate, in contrast to our findings, the JNK inhibitor SP600125 at a dose of 10 μM, used in lung explants obtained from CD1 mice at embryonic day 12.5, induced endogenous CTGF expression, TGFβ1-induced CTGF expression, increased DNA fragmentation and cleaved caspase 3 [19]. Adult JNK1 null mutant mice have been reported to have increased susceptibility to hyperoxia [6]. Interestingly, in contrast, the same group of investigators reported that adult JNK1 null mutant mice were resistant to ventilation-induced lung injury [32]. In addition, adult rats treated with a JNKi were protected from LPS-induced lung injury [33]. Hence, it is obvious that depending on the specific experimental conditions, the response to JNK inhibition can be quite variable.

In addition, another important factor to consider is the significant developmental regulation in the response of the developing lung to hyperoxia, versus the adult lung, as shown by us [34,35] and other investigators [4]. This can be the potential explanation of the seemingly conflicting results of our studies versus those in JNK1 and JNK2 null mutant adult mice having increased lethality on exposure to hyperoxia [6]. We did, however use developmentally-appropriate NB mice for our *in vivo *work, in an attempt to mimic human BPD. This brings into focus the fact that independent confirmation of findings under appropriate and clinically relevant conditions must be undertaken, instead of extrapolating from *in vitro *or adult lung experimental results [36,37].

## Conclusions

**Figure 9:**
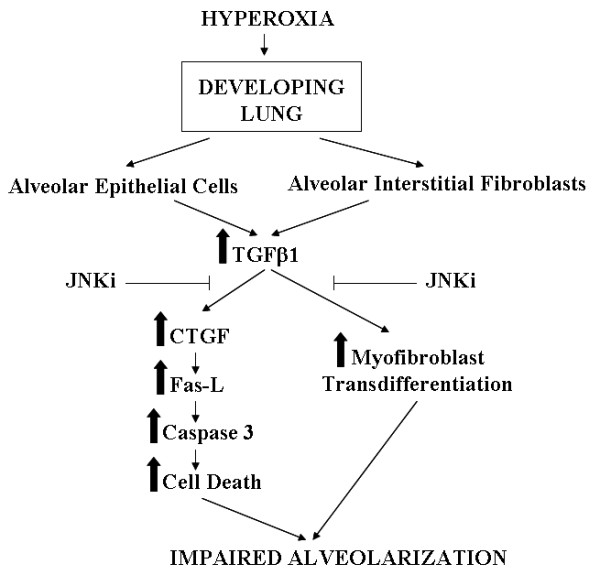
**A schematic for the proposed mechanism for the effects noted in our experimental models is shown**. Hyperoxia exposure to the developing murine lung acts on alveolar epithelial cells and interstitial fibroblasts to increase TGF-β1. In alveolar epithelial cells, this leads to increased CTGF and activation of the Fas-L and caspase-3 mediated cell death pathway. In the alveolar interstitial fibroblasts, TGF-β1 enhances myofibroblast transdifferentiation. The combined effect of increased cell death and myofibroblast transdifferentiation leads to impaired alveolarization, resulting in the pulmonary phenotype of larger, simplified alveoli, mimicking human bronchopulmonary dysplasia. JNK pathway inhibition impacts on downstream effects as shown in the figure. TGF-β1: transforming growth factor beta; JNKi: JNK inhibitor; CTGF: connective tissue growth factor; Fas-L: Fas-ligand.

A schematic for the proposed mechanism for the effects noted in our experimental models is shown as Figure 9. To summarize, our *in vitro *and *in vivo *studies demonstrate a role of the JNK pathway, at least in part, in hyperoxia-induced cell death, myofibroblast transdifferentiation, TGF-β1- and hyperoxia-mediated pulmonary responses.

## Methods

### In vitro experiments

#### Cell Culture

Human lung adenocarcinoma A549 cells were obtained from American Type Culture Collection (ATCC, Manassas, VA) and fetal rat lung (embryonic day 18) AIFs were isolated and cultured following previously described methods [20]. Cell were grown in DMEM supplemented with 10% fetal bovine serum, 100 units/ml penicillin and streptomycin (Invitrogen) and maintained at 37°C in 5% CO_2_. MLE-12 cells (ATCC, # CRL-2210, Manassas, VA) were cultured in 2% Fetal Calf Serum Dulbecco's Modified Eagle Medium, 100 units/ml penicillin and streptomycin (Invitrogen) and maintained at 37°C in humidified normoxic (95% air and 5% CO_2_) atmosphere. For different hyperoxia atmosphere (40%O_2 _and 5% CO_2_, 60%O_2_, 5% CO_2 _and 95%O_2_, 5% CO_2_) cells were placed in a sealed humidified modular incubator chamber (Billups-Rothenberg Inc.) for 24 and 48 hours time intervals. For the cell count and cell proliferation experiments, we used 6-well plates. For mRNA and protein extraction, we used 10 cm dishes. When appropriate, cells were pretreated with JNK inhibitor SP600125 (5 or 10 μM) (Calbiochem, USA) or CEP-1347 (1 μM) (a kind gift from Cephalon, West Chester, PA) for 1 h. The doses of these JNKi were based on previous reports [38-40].

#### Oxygen exposure

The cultured cells (A549, MLE and fetal rat AIFs) were then exposed to 21%O_2_, (+ 5%CO_2_) or different levels of hyperoxia (30% or 40%O_2 _+ 5%CO_2_, 60% O_2 _+ 5%CO_2_, 95%O_2 _+ 5%CO_2_). The hyperoxia group was placed in a sealed humidified hyperoxia chamber for 24 h and/or 48 h.

#### Cell Death

The viability of the cells was assessed by exclusion of trypan blue dye. At least 200 cells per experiment were counted under the microscope, and expressed as a percentage. A minimum of 3 experiments, each in duplicate, were conducted.

#### TUNEL Assay

For the quantification of undergoing apoptosis at single cell level, TUNEL assay was performed according to manufacturer's instructions (Roche Diagnostics). Cells were pretreated with and without 5 μM of JNKi, SP600125 (Calbiochem, USA) for 1 hour and subsequently exposed to normoxic or different concentrations of hyperoxia atmosphere. After treatment, cells were fixed in 4% paraformaldehyde and TUNEL-positive cells were examined at 200 × magnification using Olympus IX 70 inverted fluorescence microscope.

#### Cell Proliferation

Cell proliferation was assessed by [^3^H] thymidine incorporation assay.

#### Analysis of mRNA

RNA was isolated from A549 cells using TRIzol Reagent (Invitrogen corporation, Carlsbad, CA) according to the manufacturer's instructions. RNA samples were then DNase treated and subjected to semiquantitative RT-PCR. The primers used for semiquantitative RT-PCR: TGF-β1, 5'-TGCTCTTGTGACAGCAAAGATAA-3', 5'-CTCTGTGGAGCTGAAGCAATAGT-3', CTGF, 5'-CAAAGCAGCTGCAAATACCA-3', 5'-GGCCAAATGTGTCTTCCAGT-3', FAS, 5'-ATGCACACTCTGCGATGAAG-3', 5'-TTCAGGGTCATCCTGTCTCC-3', FAS-L 5'-CAT CAC AAC CAC TCC CAC TG-3', 5'-GTT CTG CCA GTT CCT TCT GC-3', Caspase 3, 5'-AGTCTGACTGGAAAGCCGAA-3, 5'-AAATTCTAGCTTGTGCGCGT-3'

β-actin, 5'-GTGGGCCGCTCTAGGCACCA-3', 5'-TGGCCTTAGGGTTCAGGGGG-3'. mRNA band densities were measured by densitometry and expressed in Arbitrary Densitometric Units (ADU), as previously described [34].

#### Western Blot

We detected JNK (54 and 46 kD), phospho-JNK (54 and 46 kD), FAS (48 kD), FAS-L (40 kD), Procaspase 3 (35 kD), Cleaved Caspase 3 (17 kD) protein from A549 cells lysates using Western analysis undertaken with antibodies that reacted selectively with JNK (catalog #:9252 from Cell Signaling Technology, Beverly, MA) and phospho-JNK (catalog #:V7931 from Promega Corp., Madison, WI), FAS and FAS-L (catalog #: SC7886, SC6237 from Santa Cruz Biotechnology, Santa Cruz, CA), Procaspase 3 and Cleaved Caspase 3 (catalog #: 9662, 9661 from Cell Signaling Technology, Beverly, MA), and with β-actin as control, as previously described [35]. For the AIFs, the antibodies utilized were JNK and phospho-JNK (catalog # 9252 and 9251, respectively, Cell Signaling, Danvers, MA), PPARγ (catalog # sc-7196, Santa Cruz, CA), ADRP [a gift from (late) Dr. Constantine Londos, NIDDK], fibronectin (catalog # sc-8422, Santa Cruz, CA), LEF-1 (catalog #, sc-28687, Santa Cruz, CA). Immunoreactive proteins were visualized using the Quick Spray Chemiluminescent HRP Antibody Detection Reagent (Denville Scientific, Metuchen, NJ). The membranes were exposed to HyBlot CL autoradiography film (Denville Scientific, Metuchen, NJ). Membranes were then stripped with Restore Western Blot stripping Buffer (Thermo Scientific, Rockford, IL) and re-incubated with primary antibodies reactive with β-Actin (Santa Cruz Biotechnology, Santa Cruz, CA) as normalization. The intensities of protein bands were quantified by NIH Image/ImageJ.

#### Immunohistochemistry

This was done in AIFs for Phospho-JNK detection, oil-red-O and α-SMA accordingly to previously described methods [41].

### In vivo experiments

#### Animals

TGF-β1 transgenic (TG) mice were a kind gift from Jack Elias, MD and were generated as previously described, and bred to obtain NB mice [13]. The TG mice had "human" TGF-β1 targeted to the lung using the CC10 promoter and was "turned on" with maternal exposure to doxycycline (dox) in the drinking water, leading to transmammary activation in the TG (+) pups, as described previously [13]. Activation of TGF-β1 effector pathway, as evidenced by activation of the downstream transducer, Smad2 and p-Smad2 have been previously reported in our murine model [13]. For the hyperoxia survival experiments, all NB mice (TGF-β1 TG and litter-mate WT controls) were exposed to hyperoxia and maternal dox water from postnatal day 1 (PN1) to PN7 [34,35]. For the RA experiments, all NB mice (TGF-β1 TG and litter-mate WT controls) were exposed to maternal dox or regular water from PN7 to PN10 [13]. All mice were of the C57/Bl6J strain. All animal work was approved by the Institutional Animal Care and Use Committee at the Yale University School of Medicine.

#### Oxygen exposure

For the NB animals, exposure to hyperoxia (along with their mothers) was done by placing them in cages in an airtight Plexiglass chamber (55 × 40 × 50 cm), as described previously [34,35]. For the NB survival experiments, exposure to 100% oxygen was initiated on PN1 and continued till PN7. For the NB murine model of BPD, exposure to 100% oxygen was initiated on PN1 and continued till PN4 (*vide infra*). Two lactating dams were used. They were alternated in hyperoxia and RA every 24 h. The litter size was kept limited up to 10-12 pups per dam to control for the effects of litter size on nutrition and growth.

Throughout the experiment, they were given free access to food and water. Oxygen levels were constantly monitored. The inside of the chamber was kept at atmospheric pressure, and mice were exposed to a 12 h light-dark cycle. We opened the oxygen chamber once a day to change the mothers and inject the mouse pups with the JNK inhibitor. We used the JNK inhibitor, SP600125, in the concentration of 2 μg/μl, dissolved in PBS, and used a dose of 20 mg/kg/day, given intra-peritoneally. The dose and route of administration of SP600125 was based on what has been previously reported [42-44].

For the hyperoxia survival experiments, NB TGF-β1 TG mice on dox water, with or without treatment with JNKi, were exposed to 100% O_2_. NB WT liter-mate mice on dox water, with or without treatment with JNKi, exposed to 100% O_2_, were used as controls.

#### NB Murine BPD model

NB WT mice were exposed to hyperoxia, as noted above, from PN1-4 (saccular stage of murine lung development) and allowed to recover in RA for the next 10 days. Mice were sacrificed on PN14. NB WT mice lungs at PN14 have the phenotype mimicking human BPD, as has also been reported by other investigators [45]. For the JNKi experiments, NB mice were injected daily with the JNK inhibitor (as noted above) for the 14 days.

#### Analysis of mRNA

RNA was isolated from frozen lungs using TRIzol Reagent (Invitrogen corporation, Carlsbad, CA) according to the manufacturer's instructions. RNA samples were then DNase treated and subjected to semiquantitative RT-PCR. The primers used for semiquantitative RT-PCR have been noted in the *in vitro *section of methods (*vide supra*).

mRNA band densities were measured by densitometry and expressed in Arbitrary Densitometric Units (ADU), as previously described [34].

#### Histology

Lung tissues obtained from the NB mice from the RA experiments at PN10, using a standard protocol for lung inflation (25 cm), were fixed overnight in 10% buffered formalin [46]. After washing in fresh PBS, fixed tissues were dehydrated, cleared, and embedded in paraffin by routine methods. Sections (5 μm) were collected on Superfrost Plus positively charged microscope slides (Fisher Scientific Co., Houston, Texas, USA), deparaffinized, and stained with hematoxylin & eosin, as described previously [35].

#### Lung Morphometry

Alveolar size was estimated from the mean chord length of the airspace as described previously [35]. At least three animals were studied at each time point in the presence and absence of dox water. Chord length increases with alveolar enlargement.

#### Bronchoalveolar lavage (BAL) fluid measurements

BAL fluid measurements of "human" TGF-β1 from the mice sacrificed at PN10 were done using ELISA (catalog #: DB100B; R&D Systems Inc., Minneapolis, MN), as per manufacturer's instructions.

#### Statistical analyses

Values are expressed as mean ± SEM or median (25^th ^-75^th ^centiles). Groups were compared with the Student's two-tailed unpaired *t *test (with Welch's correction for unequal variances), Mann-Whitney test or the logrank test, using GraphPad Prism 3.0 (GraphPad Software, Inc., San Diego, CA), as appropriate. A *P <*0.05 was considered statistically significant.

## Authors' contributions

ZL carried out the A549 cell culture experiments, the *in vivo *studies, analysis of RNA and western blots, densitometry and lung morphometry. RC-W participated in the *in vivo *studies, the analysis of RNA, ELISA measurements, densitometry and lung morphometry. HS participated in the analysis of RNA, western blots, and assisted with the *in vivo *experiments. AS carried out the MLE-12 cell culture experiments and the TUNEL assay. RS carried out the fetal lung fibroblasts studies, western blots and immunohistochemistry. VKR participated in the design of the study, the statistical analyses and helped to draft the manuscript. VB conceived of the overall study design and coordination, conducted the statistical analyses and helped to draft the manuscript. All authors read and approved the final manuscript.

## Supplementary Material

Additional file 1 **Figures S1-3 and Table S1**. The additional file figures S1-3 show the effect of JNK inhibition on total JNK and phosphorylated-JNK (P-JNK), cell death and cell death mediators in hyperoxia-exposed cells. The additional file Table S1 shows the numerical values of cell death in hyperoxia-exposed cells. Click here for file
