# Supplementary material for: A potential role of the JNK pathway in hyperoxia-induced cell death, myofibroblast transdifferentiation and TGF-β1-mediated injury in the developing murine lung
Source: BMC Cell Biol. 2011 Dec 15;12:54. doi: 10.1186/1471-2121-12-54 (PMC3266206; doi:10.1186/1471-2121-12-54)
Supplement: Additional file 1 — Figures S1-3 and Table S1. The additional file figures S1-3 show the effect of JNK inhibition on total JNK and phosphorylated-JNK (P-JNK), cell death and cell death mediators in hyperoxia-exposed cells. The additional file Table S1 shows the numerical values of cell death in hyperoxia-exposed cells. [file 1471-2121-12-54-S1.DOC]

**ADDITIONAL FILE FIGURE LEGENDS**

**Additional File Figure 1.** Effect of JNK inhibition on total JNK and phosphorylated-JNK (P-JNK) in hyperoxia-exposed cells. A549 cells were exposed to 21% up to 95% O_2_ and total JNK, P-JNK and β-actin proteins were assessed by Western Blot, with or without the JNKi. O_2_: oxygen; JNKi: JNK inhibitor.

**Additional File Figure 2.** Effect of JNK inhibition on cell death in hyperoxia-exposed cells. MLE-12 cells were exposed to 21% up to 95% O_2_ and cell viability (TUNEL assay) was assessed at 48h. The noted values represent assessments in a minimum of 4 measurements in each group. O_2_: oxygen; JNKi: JNK inhibitor. **P*<0.05, #*P*≤0.01, ##*P*≤0.001.

**Additional File Figure 3.** Effect of JNK inhibition on cell death mediators in hyperoxia-exposed cells. A549 cells were exposed to 21% up to 95% O_2_ and FAS, FAS-L, procaspase 3 and cleaved caspase 3 proteins were assessed by Western Blot. Independent experiments were done in the presence of the JNK pathway inhibitor. Representative western blots of FAS-L and cleaved caspase-3 are shown in the absence (**3A**) or presence (**3B**) of the JNki. The figures are illustrative of a minimum of 4 experiments. O_2_: oxygen; JNKi: JNK inhibitor.

**Additional File Figure 1.**

**Additional File Figure 2.**

**Additional File Figure 3A.**

**Additional File Figure 3B.**

**Additional File Table 1.** Effect of JNK inhibition on cell death and it’s mediators in hyperoxia-exposed cells. A549 cells were exposed to 21% up to 95% O_2_ and cell viability (trypan blue) was assessed at 24h. MLE-12 cells were exposed to 21% up to 95% O_2_ and cell viability (TUNEL assay) was assessed at 24h. The noted **mean ± sem** values (expressed as %) represent assessments in a minimum of 4 measurements in each group. For p-values, please see **Figures 1A** and **1B**, respectively. O_2_: oxygen; JNKi: JNK inhibitor.

| **Cell Type** | **21%O_2_** | **21%O_2_**  **+**  **JNKi** | **40%O_2_** | **40%O_2_**  **+**  **JNKi** | **60%O_2_** | **60%O_2_**  **+**  **JNKi** | **95%O_2_** | **95%O_2_**  **+**  **JNKi** |
| --- | --- | --- | --- | --- | --- | --- | --- | --- |
| **A549 cells (Trypan blue)** | 94.96 ± 1.03 | 95.41 ± 0.71 | 90.46 ± 1.06 | 94.15 ± 0.80 | 92.19 ± 0.49 | 94.94 ± 0.79 | 89.30 ± 1.88 | 93.82 ± 0.86 |
| **MLE-12 cells (TUNEL)** | 99.20 ± 0.30 | 98.85 ± 0.57 | 87.03 ± 3.30 | 92.94 ± 1.08 | 89.17 ± 1.40 | 93.70 ± 1.28 | 86.99 ± 1.61 | 91.14 ± 0.87 |
